# Effects of Perceived Stress on Problematic Eating: Three Parallel Moderated Mediation Models

**DOI:** 10.3390/nu17111928

**Published:** 2025-06-04

**Authors:** Haoyu Guo, Ziyi Ye, Jinfeng Han, Yijun Luo, Hong Chen

**Affiliations:** 1 Faculty of Psychology, Southwest University, Chongqing 400715, China; penttig@163.com (H.G.); hanjinfengpsy@126.com (J.H.); luoyijun@swu.edu.cn (Y.L.); 2 Key Laboratory of Cognition and Personality (SWU), Ministry of Education, Chongqing 400715, China; 3 College of Education Science, Hubei Normal University, Huangshi 435002, China; 15907290623@163.com; 4 Research Center of Psychology and Social Development, Southwest University, Chongqing 400715, China

**Keywords:** perceived stress, problematic eating, restrained eating, emotional eating, external eating, irrational health beliefs, negative coping styles

## Abstract

**Background:** Stress adversely affects health behaviors, particularly problematic eating. However, the psychological mechanisms underlying this relationship remain underexplored. This study seeks to examine the mediating role of irrational health beliefs and the moderating role of negative coping styles in the associations of perceived stress with three types of problematic eating—restrained, emotional, and external eating. **Methods**: A total of 929 emerging adults (57.8% females; mean age = 21.50 ± 2.36 years, age range = 17–35 years) participated in an online survey to provide their self-reported data. **Results:** Perceived stress was positively associated with restrained, emotional, and external eating. Irrational health beliefs partially mediated these associations, with indirect effects of 0.24, 0.40, and 0.07, respectively. Negative coping styles only moderated the associations of perceived stress with restrained eating (β = 0.05, *p* = 0.047) and emotional eating (β = 0.08, *p* = 0.001), but not external eating (β = 0.01, *p* = 0.859). **Conclusions**: Our findings suggest the effect of cognitive factors such as irrational health beliefs and negative coping styles on stress-induced eating. Interventions aimed at cognitively restructuring irrational health beliefs and raising attention on health, as well as promoting adaptive stress-coping strategies that alleviate emotional distress without compromising other aspects of health, are therefore essential.

## 1. Introduction

Problematic eating has the potential to negatively impact physical health, including precipitating and exacerbating obesity [1,2] and increasing the risk of such behavior developing into clinically diagnosed eating disorders [3,4]. Evidence suggests that negative emotions (e.g., anxiety, depression, and stress) play a contributory role in problematic eating [5,6]. Among them, stress and its association with eating have garnered growing concern in recent years [7,8]. This state variable is also considered a predictor for unhealthy eating behaviors or tendencies [9]. Understanding the mechanisms through which perceived stress affects problematic eating is of considerable significance. This study aims to construct and test three parallel moderated mediation models to investigate the “stress → eating” pathway, with a particular focus on cognitive factors that may have certain effects.

Although a universally accepted definition is lacking, problematic eating is commonly conceptualized as a non-clinical phenotype encompassing behaviors such as restrictive, emotional, and compulsive eating, as well as food stealing, hoarding, and contamination [10]. These behaviors are distinguished from eating disorders by not meeting clinical diagnostic criteria. In alignment with prior operational definitions of problematic eating [11,12,13], the current study assessed this construct using the restrained, emotional, and external eating subscales of the Dutch eating behavior questionnaire (DEBQ) [14]. These subscales, particularly restrained and emotional eating, capture the most prevalent and well-documented disordered eating patterns and have been widely validated as reliable measures in both minors [11,15,16] and adults [12,13,17,18], making them appropriate for our study focus. Plenty of research showed that perceived stress is strongly associated with problematic eating [19,20,21,22,23]. However, few studies have examined whether perceived stress affects problematic eating via health-related beliefs. Considering that health beliefs are robust predictors of health behaviors [24], exploring their role in the stress–eating relationship could provide new insights for developing targeted interventions that modify maladaptive health beliefs in managing stress-driven eating problems.

According to Rosenstock’s (1966) health belief model (HBM) [25], individuals’ perceptions of susceptibility, severity, benefits, and barriers play a critical role in explaining and predicting health behaviors [26]. Consequently, it could be reasonably inferred that one potential factor influencing healthy/unhealthy eating is health-related beliefs. From a preventive and interventional perspective, particular attention should be paid to irrational and incorrect health beliefs, as they are more likely to carry important implications for adverse health outcomes. Irrational health beliefs refer to cognitive distortions related to health that predict subsequent health behaviors [27]. Researchers found that irrational health beliefs are significant predictors of disease risk and progression in various physiological maladies, including hypertension [28], diabetes [29], and cardiovascular conditions [30]. This is mainly because irrational health beliefs are typically characterized by distorted and unreasonable negative cognitions [27] that sustain health anxiety, biasing people’s attention toward health-threatening information [31]. As such, it can be said that these beliefs exert a profound influence on individual health, which indicates the importance and necessity of further investigation.

While limited research explored the relationship between stress and irrational health beliefs [32], some found that irrational beliefs are associated with functional (dysfunctional) distress in stressful situations [33]. The findings support Ellis’s (1994) binary model of distress, which hypothesizes that rational and irrational beliefs are related separately to functional and dysfunctional distress [34]. Cognitive theories of depression similarly suggest that depression arises from unreasonable and unrealistic patterns of thinking. The evaluation of stressful events in an irrational or distorted manner predisposes people to mood disorders and behavioral dysfunctions [35,36]. Irrational health beliefs inherently embody the same cognitive distortions, simply in relation to the attitude toward health matters. Such beliefs reflected in health behaviors involving food intake may undermine the intention to resist the urge to consume unhealthy foods, exacerbating problematic eating. A direct empirical link between irrational health beliefs and eating behaviors is demonstrated by Osberg et al.’s (2008) [37] development of the irrational food beliefs scale (IFBS) and its associated measurement results, which show predictable associations of irrational food beliefs with recent weight gain, poor weight loss maintenance, and bulimic symptoms. These beliefs are also correlated positively with a myriad of mental health conditions (e.g., depression, anxiety, hostility, phobic anxiety, and so forth), but negatively with self-esteem and need for cognition, representing an influencing factor in weight loss and maintenance failure [37]. In their subsequent research, irrational food beliefs were also found to mediate the relationship between stress and bulimic symptoms [38], suggesting that such beliefs increase disordered eating as stress rises. Based on this, we assume that irrational health beliefs act as a mediator from perceived stress to problematic eating, where heightened stress exposure distorts the perception of health threats, reinforcing irrational health beliefs and ultimately contributing to problematic eating.

Stress is inextricably linked with coping styles, with it stemming from both the stressful events that we cannot avoid and the way we cope with them. In line with the transactional theory of stress and coping (TTSC) proposed by Lazarus and Folkman (1984), the emergence of stress is contingent upon two critical psychological processes: cognitive appraisal and coping [39]. In response to stressors, people’s coping strategies can be either positive (e.g., altering thoughts, seeking support, or taking action to manage or adapt to the issue) or negative (e.g., avoidance, venting, dreaming of a miracle, or self-paralysis) [40]. The function of coping is primarily to address problems or ease mood, with the outcomes exerting varying effects on individuals’ physical and mental health. While negative coping strategies could offer temporary relief from emotional distress [41,42,43], they typically fail to address the root cause of stress and are therefore unsustainable and, what is much worse, they may introduce new health risks. Common forms of negative coping styles involve alleviating distress through smoking, drinking, substance use, or food intake [40]. Stress tends to increase the consumption of palatable foods, as these items make those stressed feel a sense of “comfort” [44,45,46]. Nonetheless, over time, such a way of coping is likely to lead to obesity and metabolic dysfunction, while also making it harder to discontinue the habitual eating patterns driven by stress [47]. It could be argued that negative coping styles are vital in moderating the stress–eating relationship. People who prefer to adopt these styles are inclined to demonstrate more problematic eating. This study intends to examine the role of negative coping styles as a moderator of the process of stress-induced eating.

**Figure 1:**
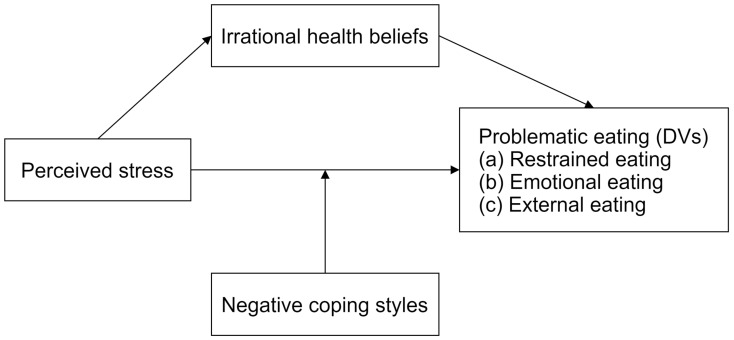
The hypothesis model of perceived stress affects three types of problematic eating (i.e., restrained, emotional, and external eating), mediated by irrational health beliefs and moderated by negative coping styles. Age, gender, education, place of birth, only child status, nationality, and BMI are controlled as covariates in the actual analysis.

Taken together, we speculated that people with more irrational health beliefs and a higher propensity for adopting negative coping styles when perceiving stress would exhibit higher levels of problematic eating. The health belief model and transactional theory of stress and coping are conceptually synergistic in explaining stress-driven eating patterns. The former focuses on why people initiate health (risk) behaviors, while the latter elucidates how these behaviors unfold through dynamic stress-coping interactions. Thus, integrating them provides a comprehensive lens to analyze moderated mediation pathways in how perceived stress affects problematic eating. Grounded in these frameworks, and in conjunction with existing findings [48,49], the current study aims to establish three parallel moderated mediation models to test the following hypotheses: (1) perceived stress is positively associated with three types of problematic eating (i.e., restrained, emotional, and external eating); (2) irrational health beliefs mediate the relationship between perceived stress and problematic eating; and (3) negative coping styles moderate the relationship between perceived stress and problematic eating (Figure 1). This work holds certain theoretical and practical value in furthering our understanding of the effects of perceived stress on problematic eating, as well as preventing and intervening in health risk behaviors resulting from inappropriate ways of coping with stress.

## 2. Materials and Methods

### 2.1. Participants and Procedure

Participants were recruited through a convenience sampling from four universities located in Chongqing and Hubei Province, China, and they completed an online questionnaire. The survey was conducted during the Spring 2024 semester. As it was administered online, each unique IP address was allowed to submit only once to prevent duplicate entries. After excluding participants with inattentive responses (e.g., excessively brief response times or selecting the same option for all items on any one of the scales), 929 (57.8% female) out of 1141 were included in the subsequent analysis. The sample was composed of emerging adults with ages ranging from 17 to 35 (*M* = 21.50, *SD* = 2.36) and body mass indices (BMIs) ranging from 13.06 to 32.60 (*M* = 20.62, *SD* = 2.61). Other sample information is shown in Table 1.

All participants accessed the Wenjuanwang (https://www.wenjuan.com, accessed on 31 May 2024) platform to fill in the questionnaire via the link or QR code we provided. Prior to the start of the test, participants were informed about the anonymous nature of the study and the adequate protection of their information. They were also permitted to withdraw at any time without the need to justify. In appreciation of the participants’ time, we paid them an honorarium (CNY 10, approximately USD 1.4), which would be provided via electronic transfer upon completion of the survey. The work conducted in this study adhered to the Declaration of Helsinki (DoH), with all procedures receiving approval from the Ethics Committee of the Faculty of Psychology at Southwest University.

### 2.2. Measures

#### 2.2.1. Perceived Stress

Perceived stress was measured using the perceived stress scale-10 (PSS-10), which was revised by Wang et al. (2011) [50]. The 10-item scale includes two dimensions: negative feelings (6 items, e.g., “In the last month, how often have you been upset because of something that happened unexpectedly?”) and positive feelings (4 items, e.g., “In the last month, how often have you confident about your ability to handle your personal problems?”), with a 5-point Likert scale for scoring that ranges from 0 (never) to 4 (very often). All items within the positive feelings dimension need to be scored in reverse. Lee et al. (2012) found that the psychometric properties of the PSS-10 are superior to its original version (i.e., PSS-14) [51,52]. The PSS-10 has been widely used for the assessment of perceived stress in the Chinese population, demonstrating good reliability and validity [53,54,55]. In the current study, the Cronbach’s α coefficient for the scale was 0.78.

#### 2.2.2. Problematic Eating

Problematic eating was measured using the Dutch Eating Behavior Questionnaire (DEBQ), which was revised by Kong (2012) [56]. The questionnaire comprises three subscales: restrained eating (10 items, e.g., “If you have put on weight, do you eat less than you usually do?”), emotional eating (13 items, e.g., “Do you have the desire to eat when you are irritated?”), and external eating (10 items, e.g., “If food tastes good to you, do you eat more than usual?”), amounting to a total of 33 items. Participants are required to respond to each item on a 5-point Likert scale ranging from 1 (never) to 5 (always). The latest revision of the questionnaire by Li et al. (2018) further indicated that the DEBQ is suitable for measurement among the Chinese population [57]. The Cronbach’s α coefficients for the restrained, emotional, and external eating subscales were 0.92, 0.95, and 0.81, respectively.

#### 2.2.3. Irrational Health Beliefs

The Irrational Health Beliefs Scale (IHBS), developed by Christensen et al. (1999) [27], is an instrument specifically designed to measure irrational health beliefs, consisting of a total of 20 items. Each item on the scale is involved with a brief vignette related to a health-related experience or situation, followed by a cognition or appraisal of the context. For example, in one scenario, several of your coworkers have come down with the flu. You hear on the news that there is a flu outbreak and that people who are in contact with infected individuals should receive immunizations to reduce their chances of becoming ill. You find yourself thinking, “I had a flu vaccination last year and got sick anyway, immunizations never do me any good.” Participants are needed to assess the degree of similarity between their own attitude and the provided and respond on a 5-point Likert scale that ranges from 1 (not at all like I would think) to 5 (almost exactly like I would think). Given the absence of prior validation of the IHBS in the Chinese population, this study tested its psychometric properties and showed ideal internal consistency (α = 0.95) and construct validity (*χ*^2^/*df* = 2.71, *p* < 0.001, RMSEA = 0.04, CFI = 0.96, TLI = 0.96, SRMR = 0.03).

#### 2.2.4. Negative Coping Styles

We used the Negative Coping Style Subscale (8 items, e.g., “Try to take a rest or vacation and put aside the problems (troubles) temporarily.”) from the Simplified Coping Style Questionnaire (SCSQ) developed by Xie (1998) [40] to measure negative coping styles. Each item is rated by participants on a 5-point Likert scale ranging from 0 (never) to 3 (very often). Xie’s (1998) research showed that the questionnaire is well-aligned with the characteristics of Chinese culture and exhibits satisfactory reliability and validity [40]. The Cronbach’s α coefficient for the subscale was 0.78.

### 2.3. Statistical Analysis

Data entry, organization, and descriptive statistical analyses were conducted using SPSS (Version 26). The PROCESS macro (Version 4.3) for SPSS [58] was used to examine the mediating role of irrational health beliefs and the moderating role of negative coping styles in the relationship between perceived stress and problematic eating. Specifically, after controlling for age, gender, education, place of birth, only child status, nationality, and BMI, three parallel moderated mediation models were constructed. In each model, perceived stress was entered as the independent variable; one of the three types of problematic eating—restrained, emotional, or external eating—as the dependent variable; irrational health beliefs as the mediator; and negative coping styles as the moderator. To test the mediating effect of irrational health beliefs, we used Model 4 of the SPSS PROCESS macro [58] and applied the bias-corrected (BC) bootstrap method with 5000 resamples to calculate the 95% confidence interval (CI). The mediating effect was considered significant if the confidence interval did not contain zero. The mediation proportion was derived by dividing the indirect effect (*a* × *b*) by the total effect (*c*). Next, to test the moderating effect, the variables were first centered, and an interaction term between perceived stress and negative coping styles was computed. Model 5 of the SPSS PROCESS macro [58] was used to verify the moderated mediation model.

## 3. Results

### 3.1. Descriptive Statistics

Table 2 presents the means, standard deviations, and correlation coefficients for age, BMI, and the main variables. All of them, except for the non-significant correlation between BMI and external eating, showed significant positive correlations (proved Hypothesis 1). Moreover, perceived stress exhibited a moderate correlation with emotional eating (*r* = 0.43, *p* < 0.001), indicating a relatively strong association between the two variables. In addition to the potential influence of age and BMI on how perceived stress affects problematic eating, other demographic variables (i.e., gender, education, place of birth, only child status, and nationality) also showed more or less significant between-group differences across the main variables (see Table 1), which implied that they are likely to confuse the results. Therefore, all demographic variables investigated in this study were controlled as covariates in subsequent analyses.

### 3.2. Common Method Bias

We first conducted Harman’s single-factor test to determine the minimum number of factors needed to explain variable variance. Results yielded nine factors with eigenvalues exceeding one, with maximum variance explained at 20.93% (<50%), indicating no notable common method bias [59]. To enhance analytical rigor, we also employed confirmatory factor analysis (CFA)-based multitrait-multimethod (MTMM) technique, which demonstrated poor single-factor model fit (*χ*^2^/*df* = 6.59, *p* < 0.001, RMSEA = 0.08, CFI = 0.54, TLI = 0.53, SRMR = 0.10) [60], complementing Harman’s test and further supporting the absence of common method bias in this study.

### 3.3. Mediation Models

**Figure 2:**
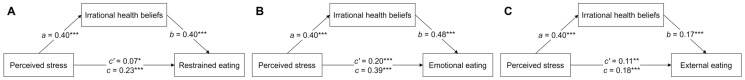
Mediation models in which irrational health beliefs mediate the associations of perceived stress with (**A**) restrained eating, (**B**) emotional eating, and (**C**) external eating. Note: * *p* < 0.05, ** *p* < 0.01, *** *p* < 0.001.

As stated above, three separate mediation models (Figure 2) were established to examine the mediating role of irrational health beliefs in the relationship between perceived stress and three types of problematic eating. Results showed that irrational health beliefs serve as a partial mediator in the associations of perceived stress with restrained eating (indirect effect = 0.24, 95% CI [0.18, 0.30], mediation proportion = 69.97%), emotional eating (indirect effect = 0.40, 95% CI [0.32, 0.48], mediation proportion = 49.32%), and external eating (indirect effect = 0.07, 95% CI [0.04, 0.11], mediation proportion = 38.92%), which provided support for Hypothesis 2.

### 3.4. Moderated Mediation Models

**Figure A1:**
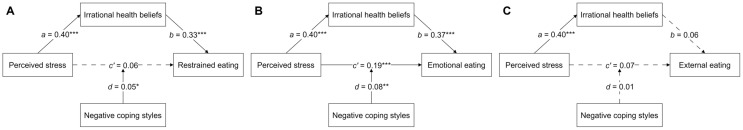
Moderated mediation models in which irrational health beliefs mediate and negative coping styles moderate the associations of perceived stress with (**A**) restrained eating, (**B**) emotional eating, and (**C**) external eating. Note: * *p* < 0.05, ** *p* < 0.01, *** *p* < 0.001.

Similarly, moderated mediation analyses were conducted for the three models (the path diagram for each outcome model is provided in Figure A1). As shown in Table 3, Table 4 and Table 5, the interaction term coefficient of perceived stress and negative coping styles is significant only in the models with restrained eating (β = 0.05, *p* = 0.047) and emotional eating (β = 0.08, *p* = 0.001) as dependent variables. In contrast, the coefficient was not significant in the model with external eating as the dependent variable (β = 0.01, *p* = 0.859). These findings indicated that negative coping styles moderate the relationship between perceived stress and restrained and emotional eating, but not external eating, partially supporting Hypothesis 3. Furthermore, we carried out a multicollinearity test on all main predictive variables and found no variance inflation factors (VIF) greater than 3 in the regression models for the three types of problematic eating, suggesting the absence of multicollinearity artifacts in the interaction terms.

**Figure 3:**
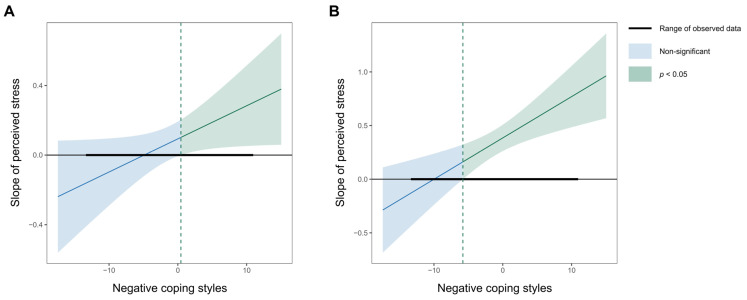
The Johnson–Neyman regions for the effect of negative coping styles on perceived stress in (**A**) restrained eating and (**B**) emotional eating. The green dashed line represents the threshold of negative coping style scores where the relationship between perceived stress and the two types of problematic eating transitions from non-significant (the blue region at the bottom-left) to significant the green region at the top-right). The shaded area around the regression line denotes the 95% confidence interval.

To further elucidate the moderated mediation model, we conducted simple slope analyses. Results indicated that for people who are more inclined to adopt negative coping styles, perceived stress significantly positively predicts restrained eating (β = 0.12, *p* = 0.015) and emotional eating (β = 0.27, *p* < 0.001), with the latter exhibiting a greater effect size. Conversely, in those adopting fewer negative coping styles, perceived stress significantly positively predicted emotional eating (β = 0.11, *p* = 0.002; with a smaller effect size compared with the high negative coping styles) but failed to predict restrained eating (β = 0.01, *p* = 0.827). The Johnson–Neyman test revealed that perceived stress is not significantly associated with restrained eating at negative coping style scores equal to or less than 0.44 (Figure 3A), or with emotional eating at scores equal to or less than −5.80 (Figure 3B). Beyond these thresholds, significant positive relationships emerged between stress and both types of problematic eating, with stronger associations as negative coping style scores rose. As these findings show, a heightened engagement in negative coping styles is related to increased restrained and emotional eating when people perceive more stress. Furthermore, negative coping styles demonstrate a more pronounced moderating effect on the relationship between perceived stress and emotional eating as opposed to restrained eating.

## 4. Discussion

The current study was designed to probe into the effect of perceived stress on problematic eating and to reveal potential mediators and moderators underlying this relationship. By doing so, we sought to deepen the understanding of the psychological mechanisms through which perceived stress affects problematic eating. As expected in Hypothesis 1, perceived stress is significantly positively associated with three types of problematic eating, aligning with previous findings [22,61,62]. We also found that the relationship between perceived stress and problematic eating is mediated by irrational health beliefs, which supports Hypothesis 2. Furthermore, some parts of Hypothesis 3 were confirmed, indicating that negative coping styles significantly positively moderate the associations of perceived stress with restrained and emotional eating, while such coping strategies fail to moderate the relationship between perceived stress and external eating. Our findings suggest that inadequate ways of coping with stress are likely to be related to new health risks, which may not only damage the individuals’ physical and mental health but also exacerbate public health challenges within society.

The Health Belief Model holds the idea that the willingness to change health behaviors comes primarily from the cognition of health, particularly beliefs about one’s own health status [25]. However, to date, no research has examined how health-related beliefs affect problematic eating induced by stress. This study successfully establishes a mediating path of “perceived stress → irrational health beliefs → problematic eating” to elucidate the significant cognitive regulatory role those unreasonable beliefs about health play in the relationship between perceived stress and problematic eating. Abnormal eating patterns under stress may occur through both emotional variables (e.g., negative emotions) [63] and cognitive variables (e.g., eating self-regulation) [64]. The emphasis on cognitive variables, particularly those associated with health, is predicated on the notion that people’s knowledge and attitudes about health can predict their engagement in health risk behaviors [65]. Therefore, on the one hand, addressing problematic eating necessitates a comprehensive enhancement of the public’s knowledge concerning balanced nutrition. On the other hand, it is essential to cultivate proactive attitudes and precaution consciousness that enable people to make timely adjustments when confronted with health-threatening conditions. These approaches would be conducive to mitigating the detrimental effects of stress and other adverse events on a healthy diet.

As has been pointed out, while the health belief model demonstrates limited predictive effect for medical adherence, it shows stronger efficacy in predicting preventive health behaviors [66]. This study was conducted based on general populations to examine problematic eating, which is non-pathological but does pose health hazards, with a focus on warning of the deterioration of such behaviors and emphasizing their preventive implications. Our findings not only reaffirm the model’s utility in predicting voluntary preventive actions but also extend its applicability to health risk behaviors manifested through problematic eating. Furthermore, for health-threatening rather than health-promoting behaviors, paying attention to their aberrant cognitive changes could inform effective prevention and intervention. Research demonstrated significant associations between cognitive distortions and various health behaviors (using negative evaluative indicators that actually measure health risk behaviors, e.g., “lack of fruits and vegetables consumption”) [67], which could substantiate the role of cognitive distortions in the occurrence and development of health risk behaviors and highlight the relevance of using irrational (rather than rational) health beliefs to predict them. Therefore, this study’s discovery suggests the importance of a key cognitive factor, irrational health beliefs, and encourages relevant practitioners to make targeted interventions that reduce problematic eating by enhancing people’s awareness of health threats and attention to health maintenance.

We also obtained intriguing and varied findings regarding the moderating role of negative coping styles. Previous studies revealed that avoidant or emotion-oriented coping styles play a mediating or moderating role in the association of perceived stress with problematic eating [23,48,61,68,69], indicating that coping strategies act as an indirect factor influencing the relationship between stress and eating [70]. According to Lazarus and Folkman (1984), coping with stress in an emotional manner mainly intends to alleviate negative emotions [39]. Emotion-focused coping styles are not identical to negative coping styles; nonetheless, both demonstrate behavioral manifestations that reflect or assuage worries (e.g., problematic eating). Xie (1998) argued that coping styles, whether positive or negative, are relative in nature, and the use of them does not necessarily bring corresponding consequences [40]. For instance, comfort eating in response to stress can relieve mental stress [71], especially when consuming foods high in sugar, fat, and calories that provide transient benefits for mood enhancement and stress reduction [72]. This may explain our findings well, suggesting that negative coping styles exert the strongest effects within models with emotional eating as the dependent variable and illustrating the immediate transactional features of “appraisal → copying → adaptation”. Additionally, given that the sample of this study comprised mainly young and normal-weight women, their restrained eating is perhaps more driven by modern Eastern society’s pursuit of the ideal body shape (e.g., media internalized pressure) [73], while stress could be addressed in this group through other coping mechanisms (e.g., emotional eating) [74], potentially attenuating the association with restrained eating. Unexpectedly, the moderating effect of negative coping styles in the model with external eating as the dependent variable was not significant. We speculate this is due to external eating relying more on environmental cues than internal signals [75]. For this type of problematic eating, situational food-cue exposure may serve as a stronger moderator. External eating demonstrated relatively lower internal consistency (α = 0.81 vs. 0.92 and 0.95), which may also reduce the power of the model. On the contrary, restrained and emotional eating pertain to self-control and emotional processing, respectively, which are closely related to negative coping styles that aim to achieve self-soothing by diverting attention away from stressors.

From cognitive appraisal theory (CAT) [39], our findings also hint at the importance of incorporating secondary appraisal and reappraisal processes in developing prevention and intervention strategies. In concrete terms, as people appraise stressors as threats and perceive inadequate coping resources, negative coping styles (e.g., problematic eating) may become a possible option for response. Consequently, promoting positive coping alternatives during the secondary appraisal could contribute to health risk avoidance. In the reappraisal phase, guiding people to evaluate and modify coping strategy efficacy, defined by sustainable mitigation of stress-related distress without long-term health compromise, proves critical. Problematic eating, while providing transient relief, constitutes a maladaptive “success” that jeopardizes long-term well-being. Thus, cognitive restructuring training appears to be of great importance, especially to enhance coping resources during secondary appraisal (e.g., mindfulness-based stress reduction and overeating improvement) [76] and to “stop the damage” during reappraisal. Notably, the transient benefits of negative coping styles suggest that interventions need to avoid simple prohibition. Drawing on dialectical behavior therapy (DBT) [77], problematic eating could be transformed into healthier alternatives (e.g., chewing sugar-free gum) to gradually achieve behavioral substitution based on recognition of the people’s current assessment level. It is essential to emphasize that these health promotion suggestions require further implementation and evaluation in both non-clinical and clinical settings to better improve human health and well-being.

While the current study offers some insights, several limitations must be acknowledged. First, this study was based on a cross-sectional design, which limited the ability for causal inferences. Also, in consideration of problematic eating itself elevating perceived stress, such as weight concerns, bidirectionality between these variables may exist. The lack of temporal sequencing restricts the interpretation of their relationship, as it is unclear whether stress contributes to problematic eating or whether these behaviors exacerbate stress over time. Future research should adopt a longitudinal survey methodology (e.g., latent growth curve model or cross-lagged panel model) to further explore the dynamics between perceived stress and problematic eating. Second, the reliance on self-report methods for data collection may be subject to social desirability bias. Given that cortisol is recognized as a biomarker of stress, which can objectively reflect stress levels [78,79,80], it is recommended that researchers employ a combined approach using both physiological and psychological indicators to measure stress in future studies. Third, as the participants were all Chinese emerging adults, caution should be exercised when generalizing our findings to other cultural contexts or age groups. Another culturally relevant concern pertains to the measurement of irrational health beliefs, as each item on the scale is presented in the form of a brief vignette. Although the scale demonstrated acceptable reliability and validity in this set of samples, its culturally embedded format necessitates further adaptation and validation within the Chinese context. Finally, this study only included restrained, emotional, and external eating as measures in accord with the operational definition of problematic eating. Other manifestations of problematic eating, such as binge eating, disinhibited eating, loss of control eating, and excessive food cravings, were not included. There is a need for the future to incorporate as many of the different facets of problematic eating as possible, to obtain more systematic and comprehensive results regarding this composite concept.

## 5. Conclusions

Problematic eating encompasses a range of eating behaviors that are irregular and harmful to health. Although these behaviors do not meet the clinical diagnostic criteria for eating disorders, they can still pose serious risks to individuals’ physical and mental health. Given the limited research that examined the role of health-related beliefs in the relationship between perceived stress and problematic eating, this study expands upon the explanatory scope of the health belief model regarding health risk behaviors manifested as problematic eating by introducing irrational health beliefs. Our findings also strengthen the evidence that negative coping styles moderate the association of stress-induced eating, suggesting the adverse effects of inappropriate coping strategies on dietary health. In sum, this work makes theoretical contributions to the psychological mechanisms of how stress affects eating behaviors, with the implications for developing more targeted educational policies and interventions to enhance stress-coping strategies that maintain good health, particularly for dietary practices.

## Figures and Tables

**Table 1:** Sample characteristics and between-group differences in main variables (*n* = 929).

Variables	*n* (%)	Perceived Stress ^a^	Restrained Eating ^a^	Emotional Eating ^a^	External Eating ^a^	Irrational Health Beliefs ^a^	Negative Coping Styles ^a^
**Gender**		3.84 ***	1.51	5.20 ***	−0.07	12.62 ***	5.60 ***
Male	392 (42.2%)						
Female	537 (57.8%)						
**Education**		0.82	1.35	0.09	1.49	4.33 *	1.47
Undergraduate	851 (91.6%)						
Master’s student	74 (8.0%)						
Doctoral student	4 (0.4%)						
**Place of birth**		2.59 *	5.07 ***	3.27 **	1.83	5.48 ***	3.37 ***
City	530 (57.1%)						
Country	399 (42.9%)						
**Only child status**		2.55 *	4.83 ***	5.23 ***	2.08 *	8.15 ***	4.25 ***
Only child	416 (44.8%)						
Non-only child	513 (55.2%)						
**Nationality**		2.73 **	2.09 *	2.42 *	1.60	2.37 *	0.62
Han	864 (93.0%)						
Minority	65 (7.0%)						

Note: ^a^ *t*-value or *F*-value based on analysis between-group differences; * *p* < 0.05, ** *p* < 0.01, *** *p* < 0.001.

**Table 2:** Descriptive statistics and correlation matrix for age, BMI, and the main variables (*n* = 929).

Variables	*M* (*SD*)	Range	1	2	3	4	5	6	7	8
1. Age	21.50 (2.36)	17−35	1							
2. BMI	20.62 (2.61)	13.06−32.60	0.08 *	1						
3. Perceived stress	19.10 (6.22)	0−37	0.16 ***	0.07 *	1					
4. Restrained eating	30.71 (9.40)	10−50	0.20 ***	0.21 ***	0.27 ***	1				
5. Emotional eating	36.64 (12.81)	13−65	0.17 ***	0.11 ***	0.43 ***	0.42 ***	1			
6. External eating	34.83 (6.54)	11−50	0.15 ***	0.02	0.20 ***	0.31 ***	0.45 ***	1		
7. Irrational health beliefs	56.13 (18.28)	21−92	0.28 ***	0.14 ***	0.47 ***	0.42 ***	0.57 ***	0.22 ***	1	
8. Negative coping styles	13.21 (4.23)	0−24	0.24 ***	0.10 **	0.40 ***	0.35 ***	0.49 ***	0.30 ***	0.57 ***	1

Note: *M* = mean, *SD* = standard deviation; * *p* < 0.05, ** *p* < 0.01, *** *p* < 0.001.

**Table 3:** Moderated mediation effects test with restrained eating as the dependent variable (*n* = 929).

Variables	Equation 1: Irrational Health Beliefs					Equation 2: Restrained Eating				
	β	*SE*	*t*	Bootstrap 95% CI		β	*SE*	*t*	Bootstrap 95% CI	
				LLCI	ULCI				LLCI	ULCI
Age	0.14	0.03	4.92 ***	0.09	0.20	0.07	0.03	2.08 *	0.00	0.13
Gender	−0.28	0.03	−9.65 ***	−0.33	−0.22	0.21	0.03	6.28 ***	0.14	0.27
Education	0.03	0.03	0.98	−0.03	0.08	−0.02	0.03	−0.65	−0.08	0.04
Place of birth	−0.04	0.03	−1.39	−0.10	0.02	−0.08	0.03	−2.51 *	−0.14	−0.02
Only child status	−0.12	0.03	−3.91 ***	−0.17	−0.06	−0.02	0.03	−0.68	−0.09	0.04
Nationality	0.00	0.03	0.05	−0.05	0.05	−0.02	0.03	−0.56	−0.07	0.04
BMI	−0.01	0.03	−0.20	−0.06	0.05	0.20	0.03	6.66 ***	0.14	0.26
Perceived stress	0.40	0.03	14.95 ***	0.35	0.45	0.06	0.03	1.84	0.00	0.13
Irrational health beliefs						0.33	0.04	8.20 ***	0.25	0.40
Negative coping styles						0.15	0.04	4.13 ***	0.08	0.22
Perceived stress × Negative coping styles						0.05	0.03	1.98 *	0.00	0.11
*R*	0.61					0.52				
*R* ^2^	0.37					0.27				
*F* (*df*)	68.14 *** (8, 920)					30.25 *** (11, 917)				

Note: BMI = body mass index; *SE* = standard error; CI = confidence interval; LLCI = lower limit confidence interval; ULCI = upper limit confidence interval. * *p* < 0.05, *** *p* < 0.001.

**Table 4:** Moderated mediation effects test with emotional eating as the dependent variable (*n* = 929).

Variables	Equation 1: Irrational Health Beliefs					Equation 2: Emotional Eating				
	β	*SE*	*t*	Bootstrap 95% CI		β	*SE*	*t*	Bootstrap 95% CI	
				LLCI	ULCI				LLCI	ULCI
Age	0.14	0.03	4.92 ***	0.09	0.20	0.00	0.03	−0.11	−0.06	0.05
Gender	−0.28	0.03	−9.65 ***	−0.33	−0.22	0.06	0.03	2.12 *	0.00	0.12
Education	0.03	0.03	0.98	−0.03	0.08	−0.04	0.03	−1.59	−0.10	0.01
Place of birth	−0.04	0.03	−1.39	−0.10	0.02	0.01	0.03	0.30	−0.05	0.06
Only child status	−0.12	0.03	−3.91 ***	−0.17	−0.06	−0.03	0.03	−0.94	−0.09	0.03
Nationality	0.00	0.03	0.05	−0.05	0.05	−0.03	0.03	−1.05	−0.08	0.02
BMI	−0.01	0.03	−0.20	−0.06	0.05	0.04	0.03	1.43	−0.01	0.09
Perceived stress	0.40	0.03	14.95 ***	0.35	0.45	0.19	0.03	6.14 ***	0.13	0.25
Irrational health beliefs						0.37	0.04	10.31 ***	0.30	0.44
Negative coping styles						0.23	0.03	7.10 ***	0.17	0.30
Perceived stress × Negative coping styles						0.08	0.02	3.25 **	0.03	0.13
*R*	0.61					0.63				
*R* ^2^	0.37					0.40				
*F* (*df*)	68.14 *** (8, 920)					55.10 *** (11, 917)				

Note: BMI = body mass index; *SE* = standard error; CI = confidence interval; LLCI = lower limit confidence interval; ULCI = upper limit confidence interval. * *p* < 0.05, ** *p* < 0.01, *** *p* < 0.001.

**Table 5:** Moderated mediation effects test with external eating as the dependent variable (*n* = 929).

Variables	Equation 1: Irrational Health Beliefs					Equation 2: External Eating				
	β	*SE*	*t*	Bootstrap 95% CI		β	*SE*	*t*	Bootstrap 95% CI	
				LLCI	ULCI				LLCI	ULCI
Age	0.14	0.03	4.92 ***	0.09	0.20	0.10	0.03	2.82 **	0.03	0.17
Gender	−0.28	0.03	−9.65 ***	−0.33	−0.22	0.10	0.04	2.76 **	0.03	0.17
Education	0.03	0.03	0.98	−0.03	0.08	−0.07	0.03	−1.99 *	−0.13	0.00
Place of birth	−0.04	0.03	−1.39	−0.10	0.02	−0.01	0.03	−0.25	−0.08	0.06
Only child status	−0.12	0.03	−3.91 ***	−0.17	−0.06	−0.02	0.04	−0.43	−0.09	0.05
Nationality	0.00	0.03	0.05	−0.05	0.05	−0.04	0.03	−1.39	−0.11	0.02
BMI	−0.01	0.03	−0.20	−0.06	0.05	0.01	0.03	0.18	−0.06	0.07
Perceived stress	0.40	0.03	14.95 ***	0.35	0.45	0.07	0.03	1.96	0.00	0.14
Irrational health beliefs						0.06	0.04	1.34	−0.03	0.14
Negative coping styles						0.23	0.04	5.81 ***	0.15	0.31
Perceived stress × Negative coping styles						0.01	0.04	0.18	−0.05	0.06
*R*	0.61					0.34				
*R* ^2^	0.37					0.12				
*F* (*df*)	68.14 *** (8, 920)					10.91 *** (11, 917)				

Note: BMI = body mass index; *SE* = standard error; CI = confidence interval; LLCI = lower limit confidence interval; ULCI = upper limit confidence interval. * *p* < 0.05, ** *p* < 0.01, *** *p* < 0.001.

## Data Availability

To protect participant privacy, the data presented in this study are not publicly available, but anonymized data can be available on request from the corresponding author.
